# Antioxidant Peptides from the Protein Hydrolysate of Spanish Mackerel (*Scomberomorous niphonius*) Muscle by in Vitro Gastrointestinal Digestion and Their In Vitro Activities

**DOI:** 10.3390/md17090531

**Published:** 2019-09-12

**Authors:** Guo-Xu Zhao, Xiu-Rong Yang, Yu-Mei Wang, Yu-Qin Zhao, Chang-Feng Chi, Bin Wang

**Affiliations:** 1 National and Provincial Joint Laboratory of Exploration and Utilization of Marine Aquatic Genetic Resources, National Engineering Research Center of Marine Facilities Aquaculture, School of Marine Science and Technology, Zhejiang Ocean University, Zhoushan 316022, China; xuzhao1109@sina.com (G.-X.Z.); yxr1948008999@163.com (X.-R.Y.); 2 Zhejiang Provincial Engineering Technology Research Center of Marine Biomedical Products, School of Food and Pharmacy, Zhejiang Ocean University, Zhoushan 316022, China; wangym731@126.com (Y.-M.W.); zhaoy@hotmail.com (Y.-Q.Z.)

**Keywords:** Spanish mackerel (*Scomberomorous niphonius*), muscle, peptide, antioxidant activity, stability

## Abstract

For the full use of Spanish mackerel (*Scomberomorous niphonius*) muscle to produce antioxidant peptides, the proteins of Spanish mackerel muscle were separately hydrolyzed under five kinds of enzymes and in vitro gastrointestinal digestion, and antioxidant peptides were isolated from the protein hydrolysate using ultrafiltration and multiple chromatography methods. The results showed that the hydrolysate (SMPH) prepared using in vitro GI digestion showed the highest degree of hydrolysis (27.45 ± 1.76%) and DPPH radical scavenging activity (52.58 ± 2.68%) at the concentration of 10 mg protein/mL among the six protein hydrolysates, and 12 peptides (SMP-1 to SMP-12) were prepared from SMPH. Among them, SMP-3, SMP-7, SMP-10, and SMP-11 showed the higher DPPH radical scavenging activities and were identified as Pro-Glu-Leu-Asp-Trp (PELDW), Trp-Pro-Asp-His-Trp (WPDHW), and Phe-Gly-Tyr-Asp-Trp-Trp (FGYDWW), and Tyr-Leu-His-Phe-Trp (YLHFW), respectively. PELDW, WPDHW, FGYDWW, and YLHFW showed high scavenging activities on DPPH radical (EC_50_ 1.53, 0.70, 0.53, and 0.97 mg/mL, respectively), hydroxyl radical (EC_50_ 1.12, 0.38, 0.26, and 0.67 mg/mL, respectively), and superoxide anion radical (EC_50_ 0.85, 0.49, 0.34, and 1.37 mg/mL, respectively). Moreover, PELDW, WPDHW, FGYDWW, and YLHFW could dose-dependently inhibit lipid peroxidation in the linoleic acid model system and protect plasmid DNA (pBR322DNA) against oxidative damage induced by H_2_O_2_ in the tested model systems. In addition, PELDW, WPDHW, FGYDWW, and YLHFW could retain their high activities when they were treated under a low temperature (<60 °C) and a moderate pH environment (pH 5–9). These present results indicate that the protein hydrolysate, fractions, and isolated peptides from Spanish mackerel muscle have strong antioxidant activity and might have the potential to be used in health food products.

## 1. Introduction

Food nutrition is intricately linked with human health because they can provide the necessary bioactive substances and cause specific physiological responses in the human body [1]. Among all the biological nutrients, food proteins, hydrolysates, and peptides are believed to the most well researched biomolecules [1,2]. Bioactive peptides are encrypted in the protein sequences and released by the hydrolysis action of proteases or fermentation [3,4]. Over the last decade, there has been an explosion of scientific research on the topic of bioactive peptides, which display a broad scope of functions beyond basic nutritional benefits, such as antioxidant, immunomodulatory, antihypertensive, metal-chelation, cytomodulatory, antimicrobial, antithrombotic, and opiate activities [1,5,6]. Therefore, bioactive peptides have attracted a high amount of interest from researchers and consumers because of their huge potential of serving as functional components applied in foods and other dietary supplements [3,7].

Recently, antioxidant peptides (APs) from food resources, especially from aquatic products and their byproducts, have caused widespread attention because of their safety and strong capacities in regard to reactive oxygen species (ROS) scavenging, DNA protection, and lipid peroxidation inhibition [3,8,9,10]. Moreover, seafood-derived APs could upregulate the level of intracellular antioxidant enzymes, such as superoxide dismutase (SOD), catalase (CAT), glutathione (GSH) peroxidase (GSH-Px), and GSH reductase (GSH-Rx), to protect cells and organism from the damage of oxidative stress [3,11,12]. YGDEY isolated from the gelatin hydrolysate of tilapia skin could effectively prevent UVB-induced photoaging in human keratinocytes (HaCaT) cells through decreasing levels of intracellular ROS, MMP-1 (collagenase), and MMP-9 (gelatinase), increasing antioxidant factor (SOD and GSH) expression and type I procollagen production, maintaining a balance between GSH and GSSG, and preventing DNA from oxidative damage [13]. You et al. reported that loach peptide (500 < MW < 1000 Da) prepared using flavorzyme digestion could effectively increase the swimming time of mice and decrease levels of blood urea nitrogen (BUN) and liver malonaldehyde (MDA) in mice [14]. Himaya et al. reported that GGFDMG from the gelatin hydrolysate of Japanese flounder skin could protect leukemia cells in mouse macrophage (RAW 264.7) from ROS-mediated intracellular macromolecule damage through scavenging intracellular ROS by upregulating the expression levels of inherent antioxidative factors (SOD-1, GSH, and CAT) [15]. Lin et al. indicated that the gill hydrolysate of bighead carp had high Fe^2+^-chelating and 2,2-diphenyl-1-picrylhydrazyl (DPPH) radical (DPPH·) scavenging activity. In addition, surimi with the gill hydrolysate had greater Ca^2+^-ATPase activity, higher salt-soluble and sulfhydryl protein concentrations, lower disulfide bonds, carbonyls, and hydrophobicity, as well as better gel strength and texture [16]. Therefore, APs from food resources, especially from seafoods and their byproducts, have huge potential for use in functional foods and other dietary interventions of food preservation, disease control, and health promotion.

Spanish mackerel (*Scomberomorous niphonius*) is a subset of the mackerel family (Scombridae) and distributed in the Western North Pacific, including the East China Sea, the Yellow Sea, and the Bohai Sea of China. Recently, some bioactive ingredients have been prepared and identified from the skins and bones of Spanish mackerel [17]. Li et al. isolated acid and pepsin soluble collagens from Spanish mackerel skins and bones and characterized them as type I collagen [17]. Subsequently, the skin collagen hydrolysate and fractions of Spanish mackerel were prepared, and they showed strong antioxidant activities [18]. In addition, eight APs including GPY, GPTGE, PFGPD, GPTGAKG, PYGAKG, GATGPQG, GPFGPM, and YGPM were isolated from the skin collagen hydrolysate fraction (F7) [6]. Among them, PFGPD, PYGAKG, and YGPM could effectively inhibit lipid peroxidation, reduce Fe^3+^ to Fe^2+^, and scavenge DPPH·, hydroxyl radical (HO·), superoxide anion radical ($O_{2}^{-}$·), and 2,2′-azino-bis(3-ethylbenzothiazoline-6-sulphonic acid (ABTS) cation radical in a concentration-activity manner. However, no literature regarding APs from Spanish mackerel muscle has been reported. Thus, the objectives of this paper are to (i) isolate and characterize APs from protein hydrolysate of Spanish mackerel muscle by in vitro gastrointestinal (GI) digestion and (ii) evaluate the in vitro antioxidant and stability properties of the isolated APs.

## 2. Results and Discussion

### 2.1. Preparation of Protein Hydrolysate of Spanish Mackerel (S. niphonius) Muscle

The defatted Spanish mackerel muscles were separately hydrolyzed under five kinds of enzymes and in vitro GI digestion (pepsin-trypsin system). As shown in Table 1, the protein hydrolysate (SMPH) prepared using in vitro GI digestion showed the highest degree of hydrolysis (DH, 27.45 ± 1.76%) among the six protein hydrolysates. Similarly, DPPH· scavenging activity of SMPH (52.58 ± 2.68%) was significantly higher than those of the crude protein of defatted muscle (SMP) (14.26 ± 1.03%) and protein hydrolysates using pepsin (27.64 ± 1.48%), neutrase (34.28 ± 1.37%), papain (25.98 ± 1.55%), trypsin (32.96 ± 2.33%), and alcalase (41.53 ± 3.41%), respectively (*p* < 0.05).

The specificity of the protease applied for the hydrolysis process is the key factor for the production of APs because the protein hydrolysates displayed very different spectra of substrate specificity, such as DH, biological activity, and nutritive values [3]. Wang et al. reported that the neutrase hydrolysate of blue mussel (*Mytilus edulis*) protein showed the highest DPPH· scavenging activity compared to the hydrolysates prepared using alcalase, neutrase, pepsin, and papain [9]. Agrawal et al. reported that the DH (17.47 ± 0.63%) of trypsin hydrolysate of finger millet protein was higher than that of the pepsin hydrolysate (13.73 ± 0.18%) [19]. The EC_50_ value (0.945 mg/mL) of papain hydrolysate of purple sea urchin (*Strongylocentrotus nudus*) gonad on DPPH· was significantly higher than those of trypsin (1.699 mg/mL) and dual-enzymatic (papain + trypsin) hydrolysates (2.481 mg/mL) [20]. Fish gelatin hydrolysate (FSGH) of Nile tilapia skin using ginger protease exhibited higher DH (13.08%), lipid peroxidation (48.46%), and DPPH· scavenging activity (97.21%) than hydrolysate using pepsin-pancreatin did [21]. Therefore, the protein hydrolysate (SMPH) of Spanish mackerel muscles prepared using in vitro GI digestion showed the highest DH and DPPH· scavenging activity and was chosen for further experiment.

### 2.2. Purification of APs from SMPH

#### 2.2.1. Fractionation of SMPH Using Membrane Ultrafiltration

**Figure 1:**
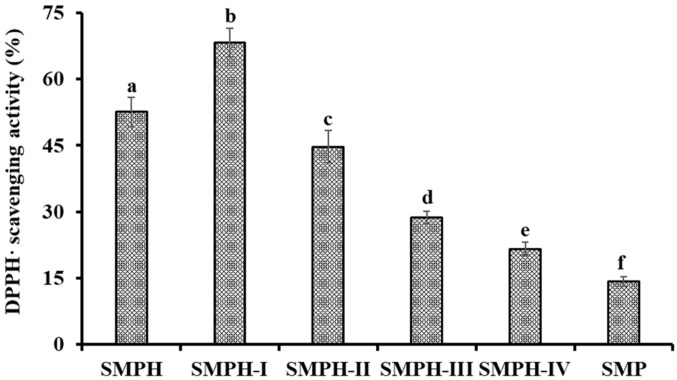
DPPH· scavenging activity of SMPH and its fractions by ultrafiltration at the concentration of 10.0 mg protein/mL. All data are presented as the mean ± SD (*n* = 3). ^a–e^ Values with the same superscripts indicate no significant difference (*p* > 0.05).

SMPH was fractionated gradually by molecular weight (MW) Cut Off (MWCO) membranes of 3, 5, and 10 kDa, and four fractions including SMPH-I (MW < 3 kDa), SMPH-II (3 kDa < MW < 5 kDa), SMPH-III (5 kDa < MW < 10 kDa), and SMPH-II (>10 kDa) were prepared. As shown in Figure 1, DPPH· scavenging activity of SMPH-I was 68.24 ± 3.29% at the concentration of 10.0 mg protein/mL, and this was significantly higher than those of SMP (14.26 ± 1.03%), SMPH (52.58 ± 2.68%), SMPH-II (44.68 ± 3.65%), SMPH-III (28.74 ± 1.41%), and SMPH-IV (21.62 ± 1.52%), respectively (*p* < 0.05). The differences in the activities among SMPH and its fractions are mainly because of the chain length, and the amino acid composition and sequence, which led to the diversity in the mechanisms of action [3]. The data agreed with the reports that the MW distribution of protein hydrolysates was negative relative to their antioxidant activity [18,22]. In addition, the lowest MW fractions from protein hydrolysates of blue-spotted stingray [23], buffalo and bovine casein [24], and *Tergillarca granosa* [25] showed the highest antioxidant activity. Therefore, SMPH-I with small MW was selected for the subsequent separation.

#### 2.2.2. Anion-Exchange Chromatography of SMPH-I

**Figure 2:**
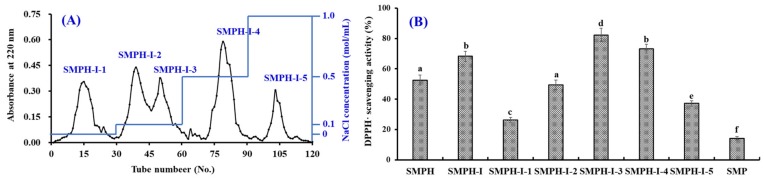
Elution profile of SMPH-I in DEAE-52 cellulose anion-exchange chromatography (**A**) and DPPH· scavenging activity of SMPH-I and its fractions at the concentration of 10.0 mg protein/mL (**B**). All data are presented as the mean ± SD (*n* = 3). ^a–f^ Values with the same superscripts of this type indicate no significant difference (*p* > 0.05).

According to the interaction strength between DEAE-52 cellulose and the hydrophobic/acidic amino acid residues in peptide sequences, five fractions (SMPH-I-1 to SMPH-I-5) were separated from SMPH-I fraction (Figure 2A). Amongst those fractions, SMPH-I-1 was eluted using deionized water (DW), SMPH-I-2 and SMPH-I-3 were eluted using 0.1 M NaCl, SMPH-I-4 was eluted using 0.5 M NaCl, and SMPH-I-5 was eluted using 1.0 M NaCl. DPPH· scavenging activities of SMPH-I and five fractions are shown in Figure 2B, and the data indicate that the DPPH· scavenging activity of SMPH-I-3 was 82.29 ± 4.37% at the concentration of 10.0 mg protein/mL, which was significantly higher than those of SMP (14.26 ± 1.03%), SMPH-I (68.24 ± 3.28%), SMPH-I-1 (26.35 ± 1.67%), SMPH-I-2 (49.43 ± 3.25%), SMPH-I-4 (73.11 ± 2.98%), and SMPH-I-5 (37.21 ± 1.69%), respectively (*p* < 0.05). Therefore, SMPH-I-3 was selected for the following experiment.

#### 2.2.3. Gel Filtration Chromatography of SMPH-I-3

**Figure 3:**
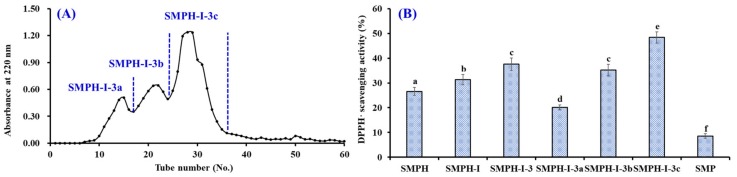
Elution profile of SMPH-I-3 in Sephadex G-25 chromatography (**A**) and DPPH· scavenging activities of SMPH-I-3 and its fractions at 5.0 mg protein/mL concentration (**B**). All data are presented as the mean ± SD of triplicate results. ^a–f^ Values with the same superscripts indicate no significant difference (*p* > 0.05).

APs separated by gel filtration depend on their molecular size, which does not directly influence their structures and bioactivities [3,6]. Therefore, gel filtration chromatography has become a popular method to concentrate and fractionate APs from different protein hydrolysates, such as croaker muscle [26], flounder fish [27], purple sea urchin gonad [20], hairtail muscle [28], and blue-spotted stingray [23]. As shown in Figure 3A, SMPH-I-3 was separated into three fractions (SMPH-I-3a, SMPH-I-3b, and SMPH-I-3c) using a Sephadex G-25 column. Figure 3B indicates that the DPPH· scavenging activity of SMPH-I-3c was 48.36 ± 2.28% at the concentration of 5.0 mg protein/mL, which was significantly higher than those of SMP (8.57 ± 0.95%), SMPH-I (31.29 ± 2.05%), SMPH-I-3 (31.29 ± 2.05%), SMPH-I-3a (20.15 ± 0.98%), and SMPH-I-3a (35.24 ± 2.31%) (*p* < 0.05). Therefore, SMPH-I-3c was selected for the following isolation process.

#### 2.2.4. Isolation of APs from SMPH-I-3c by RP-HPLC

**Figure 4:**
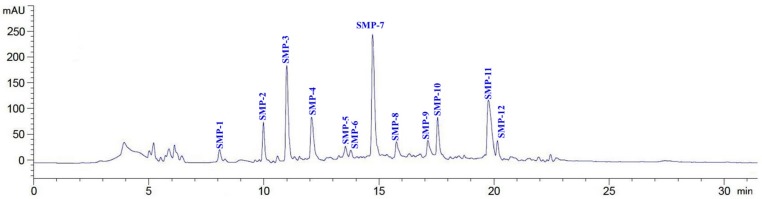
Elution profile of SMPH-I-3c separated by RP-HPLC system on a Zorbax, SB C-18 column (4.6 × 250 mm) from 0 to 30 min.

**Figure 5:**
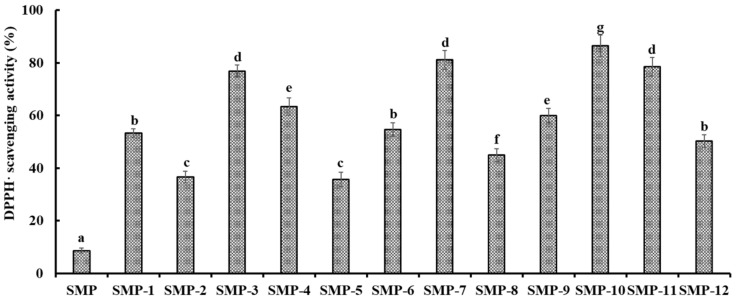
DPPH· scavenging activities of twelve major sub-fractions (SMP-1 to SMP-12) of SMPH-I-3c at the concentration of 5.0 mg protein/mL. All data are presented as the mean ± SD (*n* = 3). ^a–g^ Values with the same superscripts indicate no significant difference (*p* > 0.05).

As shown in Figure 4, 12 major peaks (SMP-1 to SMP-12) were isolated from SMPH-I-3c using the RP-HPLC system on their retention time (RT), and their DPPH· scavenging activities are shown in Figure 5. The data indicate that the DPPH· scavenging activities of SMP-3 (76.91 ± 2.36%), SMP-7 (81.09 ± 3.56%), SMP-10 (86.52 ± 4.06%), and SMP-11 (78.54 ± 3.55%) at the concentration of 5.0 mg protein/mL were significantly higher than those of other eight APs. Therefore, SMP-3, SMP-7, SMP-10, and SMP-11 with retention times of 11.02, 14.74, 17.58, and 19.83 min, respectively, were collected and lyophilized for amino acid sequence identification and activity evaluation.

### 2.3. Amino Acid Sequence and Molecular Mass Analysis of APs

**Figure 6:**
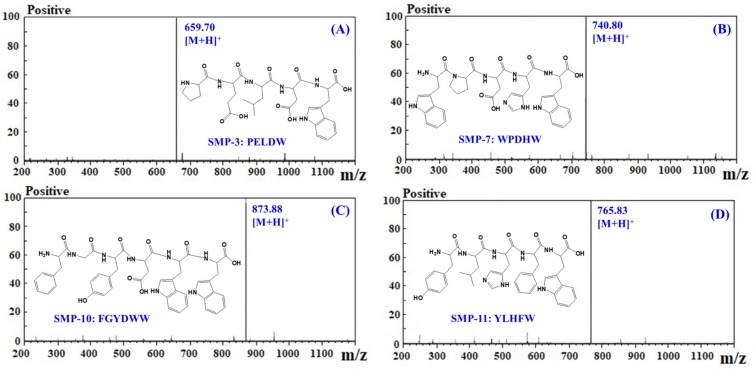
Mass spectra of four APs (SMP-3 (**A**), SMP-7 (**B**), SMP-10 (**C**), and SMP-11 (**D**)) from protein hydrolysate of Spanish mackerel (*S. niphonius*) muscle.

The amino acid sequences and molecular mass of four APs (SMP-3, SMP-7, SMP-10, and SMP-11) were determined using a protein sequencer and a quadrupole time-of-flight mass spectrometer (MS) coupled with an electrospray ionization (ESI) source, and the results are shown in Table 2 and Figure 6. The amino acid sequences of four APs were identified as Pro-Glu-Leu-Asp-Trp (PELDW, SMP-3), Trp-Pro-Asp-His-Trp (WPDHW, SMP-7), Phe-Gly-Tyr-Asp-Trp-Trp (FGYDWW, SMP-10), and Tyr-Leu-His-Phe-Trp (YLHFW, SMP-11). The detected MWs of SMP-3, SMP-7, SMP-10, and SMP-11 agreed well with their theoretical masses (Table 2).

### 2.4. Antioxidant Activity

**Figure 7:**
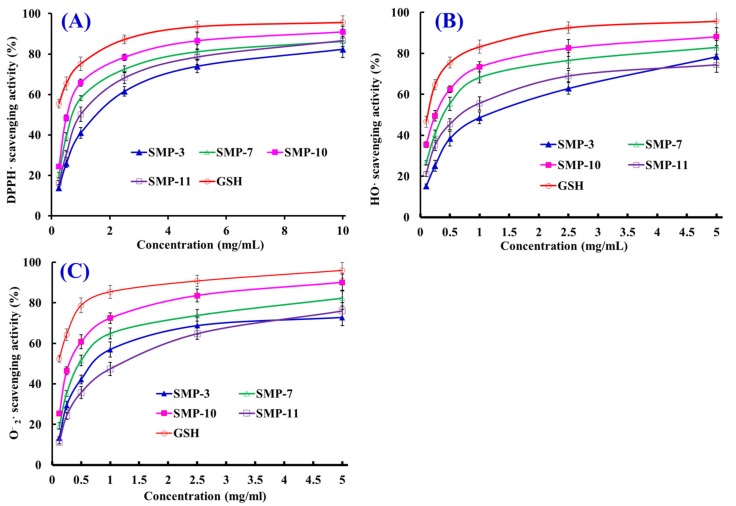
DPPH· (**A**), HO· (**B**), and $O_{2}^{-}$· (**C**) scavenging activities of four APs (SMP-3, SMP-7, SMP-10, and SMP-11) from protein hydrolysate of Spanish mackerel (*S. niphonius*) muscle. Glutathione (GSH) was used as the positive control. All data are presented as the mean ± SD (*n* = 3).

**Figure 8:**
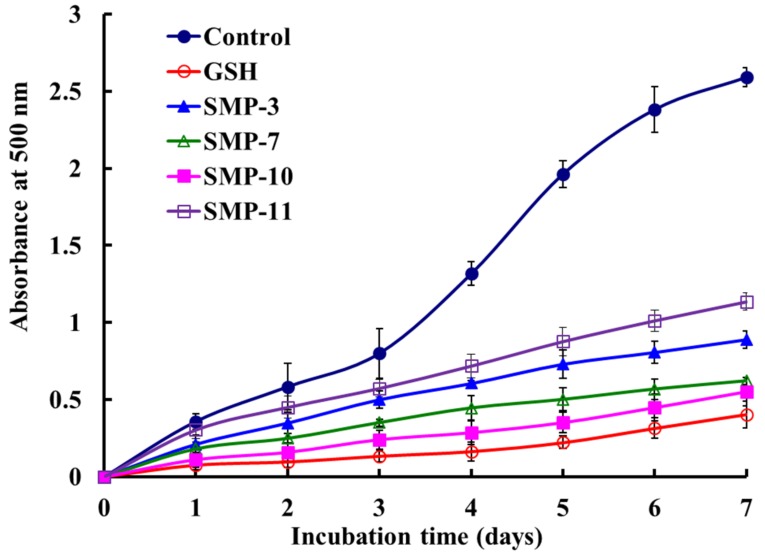
Lipid peroxidation inhibition activities of four APs (SMP-3, SMP-7, SMP-10, and SMP-11) from protein hydrolysate of Spanish mackerel (*S. niphonius*) muscle. Glutathione (GSH) was used as the positive control, and a solution without APs was used as the negative control. All data are presented as the mean ± SD (*n* = 3).

**Figure 9:**
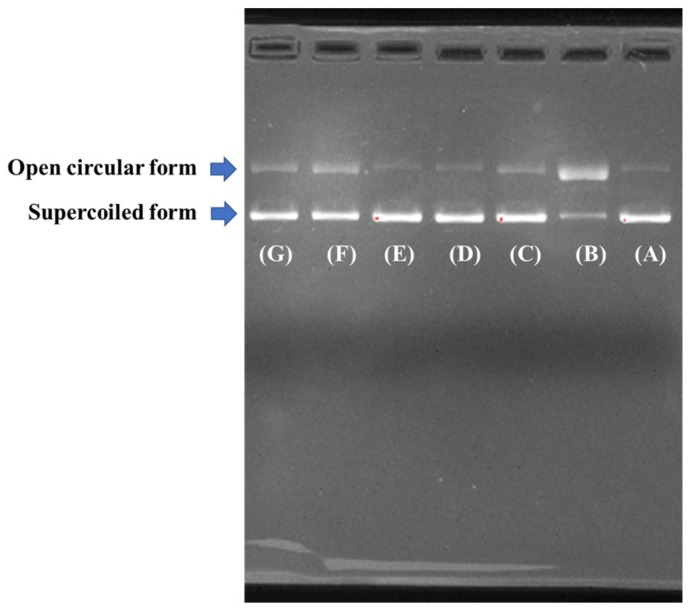
Protective effects of four APs (SMP-3, SMP-7, SMP-10, and SMP-11) on plasmid DNA damaged by H_2_O_2_. (**A**) The native pBR322DNA; (**B**) pBR322DNA treated with FeSO_4_ and H_2_O_2_; (**C**) pBR322DNA treated with FeSO_4_, H_2_O_2_, and the positive control of glutathione (GSH) (1.0 mg/mL); (**D**) pBR322DNA treated with FeSO_4_, H_2_O_2_, and SMP-11 (3.0 mg/mL); (**E**) pBR322DNA treated with FeSO_4_, H_2_O_2_, and SMP-10 (3.0 mg/mL); (**F**) pBR322DNA treated with FeSO_4_, H_2_O_2_, and SMP-7 (3.0 mg/mL); (**G**) pBR322DNA treated with FeSO_4_, H_2_O_2_, and SMP-3 (3.0 mg/mL).

Three kinds of radical (DPPH·, HO·, and $O_{2}^{-}$·) scavenging, lipid peroxidation inhibition, and plasmid DNA protective assays were used to evaluate the activity of four APs (SMP-3, SMP-7, SMP-10, and SMP-11), and the results are presented in Table 3 and Figure 7, Figure 8 and Figure 9.

#### 2.4.1. Radical Scavenging Activity

##### DPPH· Scavenging Activity

As shown in Figure 7A, four APs (SMP-3, SMP-7, SMP-10, and SMP-11) could dose-dependently scavenge DPPH· when the concentration ranged from 0.25 to 10.0 mg/mL. The half elimination ratio (EC_50_) values of SMP-3, SMP-7, SMP-10, and SMP-11 were 1.53, 0.70, 0.53, and 0.97 mg/mL, respectively, which were less effective than the positive control of GSH (0.22 mg/mL) (*p* < 0.05) (Table 3). The EC_50_ value of SMP-10 was significantly lower than those of SMP-3, SMP-7, SMP-11, and other APs from the protein hydrolysates of *Tergillarca granosa* muscle (MDLFTE: 0.53 mg/mL; WPPD: 0.36 mg/mL) [25], red stingray cartilages (IEPH: 1.90 mg/mL; LEEEE: 3.69 mg/mL; IEEEQ: 4.01 mg/mL; VPR: 4.61 mg/mL) [28], loach (PSYV: 17.0 mg/mL) [14], spotless smoothhound cartilages (GAERP: 3.73 mg/mL; GEREANVM: 1.87 mg/mL; AEVG: 2.30 mg/mL) [11], salmon pectoral fin (TTANIEDRR: 2.50 mg/mL) [29], Spanish mackerel skins (PFGPD: 0.80 mg/mL; PYGAKG: 3.02 mg/mL; YGPM: 0.72 mg/mL) [6], croceine croaker scales (GFRGTIGLVG: 1.271 mg/mL; GPAGPAG: 0.675 mg/mL) [30], *Sphyrna lewini* muscle (WDR: 3.63 mg/mL; PYFNK: 4.11 mg/mL) [31], and skipjack tuna bones (GADIVA: 0.57 mg/mL) [10]. Therefore, four APs (SMP-3, SMP-7, SMP-10, and SMP-11) could act as a contributor of electrons or hydrogen radicals to strongly inhibit the DPPH· reaction.

##### HO· Scavenging Activity

The scavenging activities of SMP-3, SMP-7, SMP-10, and SMP-11 on HO· are presented in Figure 7B and Table 3. The data indicate that SMP-3, SMP-7, SMP-10, and SMP-11 could effectively scavenge HO· in a concentration-dependent manner. The EC_50_ value of SMP-10 was 0.26 mg/mL, which was significantly lower than those of SMP-3 (1.12 mg/mL), SMP-7 (0.38 mg/mL), and SMP-11 (0.67 mg/mL), respectively, but significantly higher than that of GSH (0.12 mg/mL). Moreover, the EC_50_ value of SMP-10 was less than those of APs from croceine croaker scales (GFRGTIGLVG: 0.29 mg/mL) [30], weatherfish loach (PSYV: 2.64 mg/mL) [14], hairtail muscle (KA: 1.74 mg/mL; AKG: 2.38 mg/mL; IYG: 2.50 mg/mL) [32], grass carp skin (PYSFK: 2.283mg/mL; VGGRP: 2.055 mg/mL) [33], red stingray cartilages (VPR: 0.77 mg/mL; IEPH: 0.46 mg/mL; LEEEE: 0.70 mg/mL; IEEEQ: 1.30 mg/mL) [28], bluefin leatherjacket heads (GPP: 2.385 mg/mL; WEGPK: 5.567 mg/mL; GVPLT: 4.149 mg/mL) [8], Spanish mackerel skins (PFGPD: 0.81 mg/mL, PYGAKG: 0.66 mg/mL, and YGPM: 0.88 mg/mL) [6], and spotless smoothhound cartilages (GEREANVM: 0.34 mg/mL) [11] and muscle (GVV: 1.63 mg/mL; GFVG: 0.89 mg/mL) [34]. Superfluous HO· generated from the decomposition of hydroperoxides have highly destructive effects on key biological macromolecules and cause serial chronic diseases related to oxidative stress in organisms [4,9]. The present results indicate that four APs (SMP-3, SMP-7, SMP-10, and SMP-11) might be used as HO· scavenging agent to help the organisms from the damage of oxidative stress.

##### $O_{2}^{-}$· Scavenging Activity

Figure 7C indicates the $O_{2}^{-}$· scavenging activities of four APs (SMP-3, SMP-7, SMP-10, and SMP-11) increased significantly when their concentrations increased from 0.125 to 5.0 mg/mL, but their activities were less than that of GSH at the same concentration. The EC_50_ values of SMP-3, SMP-7, SMP-10, and SMP-11 were 0.85, 0.49, 0.34, and 1.37 mg/mL, respectively. The EC_50_ value of SMP-10 was significantly less than those of SMP-3, SMP-7, SMP-11 and other APs from skipjack tuna bones (GADIVA: 0.52 mg/mL) [25], Spanish mackerel skins (PFGPD: 0.91 mg/mL; PYGAKG: 0.80 mg/mL; YGPM: 0.73 mg/mL) [6], giant squid (LNGLEGLA: 0.864 mg/mL; NGLEGLK: 0.419 mg/mL) [35], bluefin leatherjacket heads (WEGPK: 3.223 mg/mL; GPP: 4.668 mg/mL; GVPLT: 2.8819 mg/mL) [8], hairtail muscle (KA: 2.08 mg/mL; AKG: 2.54 mg/mL; IYG: 1.36 mg/mL) [32], miiuy croaker swim bladders (YLPYA:3.61 mg/mL; VPDDD:4.11 mg/mL) [4], spotless smoothhound muscle (GVV: 0.67 mg/mL) [34], and croceine croaker scales (GFRGTIGLVG: 0.46 mg/mL) [30] and muscle (VLYEE: 0.693 mg/mL; MILMR: 0.993 mg/mL) [36]. Under harmful environmental factors, such as pollutants, γ-radiation, cigarette smoke, and UV light, the organisms will generate excessive $O_{2}^{-}$· and further be translated into HO· and peroxy radicals, which will destroy cytomembrane and key biomolecules [2,37]. Then, SMP-3, SMP-7, SMP-10, and SMP-11 can assist SOD in scavenging excess $O_{2}^{-}$· in biological systems.

#### 2.4.2. Lipid Peroxidation Inhibition Activity

The inhibiting abilities of SMP-3, SMP-7, SMP-10, and SMP-11 on the lipid peroxidation system were expressed as the absorbance of 500 nm, and the higher absorbance of the sample group illustrated lower antioxidant capacity [31]. Figure 8 shows that the absorbance of the SMP-10 group was significantly lower than those of SMP-3, SMP-7, SMP-11, and the negative control (without antioxidant), but slightly higher than that of the positive control of GSH. The data indicate that SMP-10 had the highest ability of lipid peroxidation inhibition among four APs.

The peroxidation of the membrane lipids caused by ROS can lead to cell injury and eventually unprogrammed apoptosis, and it is a crucial step in the pathogenesis of several disease states in adult and infant patients [38,39,40]. In addition, lipid peroxidation is also an important factor in high-fat food spoilage [28,36]. Therefore, the lipid peroxidation inhibition assay in the linoleic acid model system has been widely applied to evaluate the comprehensive ability of APs from seafoods, such as bluefin leatherjacket [8,36], miiuy croaker [4,7], red stingray [28], and monkfish [41]. SMP-10 can dramatically inhibit the peroxidation of linoleic acid over 7 days of incubation and has significant potential applications in food and medicine.

#### 2.4.3. Protective Effect on Plasmid DNA Damaged by H_2_O_2_

In the assay, HO· was produced from the decomposition of H_2_O_2_ mediated by iron when FeSO_4_ and H_2_O_2_ were added to the sample solutions, and the resulted HO· subsequently broke the supercoiled DNA and converted the supercoiled form into the open circular and/or linear form. Therefore, the protective effects of four APs (SMP-3, SMP-7, SMP-10, and SMP-11) on the oxidative damage of pBR322DNA induced by H_2_O_2_ were measured, and the results are shown in Figure 9. The results indicate that the plasmid DNA (pBR322DNA) was mainly of the supercoiled form under normal conditions (Figure 9A). An open circular form was generated when one phosphodiester chain of a supercoiled form of plasmid DNA was broken by HO· (Figure 9B). However, almost no linear form of DNA was found in Figure 9B, which indicates that the HO· produced from iron-mediated decomposition of H_2_O_2_ might be too little to break some double-strand of DNA in the assay. As shown in Figure 9C-E, the contents of the open circular form of DNA was obvious lower than that of Figure 9B, which indicates that four APs (SMP-3, SMP-7, SMP-10, and SMP-11) and the positive control of GSH have different protective effects on DNA damaged by oxidation, and the protective effect of SMP-10 was slightly higher than that of SMP-7 and significantly higher than those of SMP-3 and SMP-7. In addition, the image of SMP-10 and SMP-7 was similar to those of the positive control of GSH (Figure 9C) and the normal control (Figure 9A). Therefore, four APs (SMP-3, SMP-7, SMP-10, and SMP-11), especially SMP-10 and SMP-11, have high abilities to guard the supercoiled pBR322DNA against HO·-dependent strand breaks. In the organism, DNA damage is a key step in ROS-induced degenerative processes, such as premature aging, hepatopathy, and diabetes, cancer, atherosclerosis, and neurodegenerative diseases [42,43]. The present results indicate that SMP-10 had a potential ability to protect pBR322DNA from oxidative damage, and our future experiment will be performed on the cell and in vivo.

### 2.5. Effects of Thermal and pH Treatments on the Stability of SMP-3, SMP-7, SMP-10, and SMP-11

**Figure 10:**
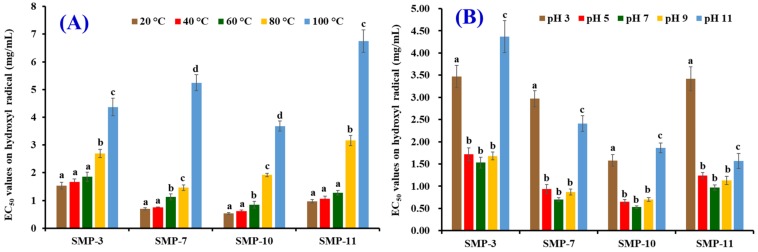
EC_50_ values of SMP-3, SMP-7, SMP-10, and SMP-11 on HO· scavenging activities when they were treated at different temperatures (**A**) and pH values (**B**). All data are expressed as mean ± SD (*n* = 3). ^a–d^ Values with the same letters indicate no significant difference of same sample (*p* > 0.05).

Figure 10A shows that the effects of temperature on HO· scavenging activity of SMP-3, SMP-7, SMP-10, and SMP-11 (expressed as an EC_50_ value). No significant difference in EC_50_ values of SMP-3 and SMP-11 was found when the treated temperature was 20, 40, and 60 °C (*p* > 0.05), but their EC_50_ values significantly increased when the treated temperatures increased to 80 and 100 °C (*p* < 0.05). Compared with SMP-3 and SMP-11, thermal treatment had stronger effects on SMP-7 and SMP-10 because their EC_50_ values treated at 60 °C were significantly (*p* > 0.05) higher than those of SMP-7 and SMP-10 treated at 20 and 40 °C (*p* < 0.05). The results indicate that SMP-3 and SMP-11 could retain their antioxidant activity when the treated temperature was lower than 60 °C, but SMP-7 and SMP-10 would lose their function at the same processing temperature. Figure 10B shows the EC_50_ values of SMP-3, SMP-7, SMP-10, and SMP-11 on HO· when they were treated at a pH value ranging from 3 to 11. No significant difference on EC_50_ values of SMP-3, SMP-7, SMP-10, or SMP-11 was found when pH value ranged from 5 to 9, but pH values of 3 and 11 significantly affected the EC_50_ values of SMP-3, SMP-7, SMP-10, and SMP-11 (*p* < 0.05).

Thermal and pH treatments are popular processing methods of food products for altering their taste, physicochemical properties, nutritional ingredients, and safety. Therefore, the stability of APs on thermal and pH treatments is closely related to their application scopes [32,44,45]. Thermal treatment can eliminate the majority of spoilage and pathogenic microorganisms, and APs can effectively inhibit lipid peroxidation if they have strong heat-resistant properties. A combination of APs and heat treatment will significantly prolong the shelf life of products. In addition, APs with broad acid-alkali tolerance properties can be used in more food products [23]. Two antioxidant hexapeptides (WAFAPA and MYPGLA) from the hydrolysate of blue-spotted stingray showed high stability because their EC_50_ values on HO· were not significantly different when they were treated at 25–100 °C or at pH values of 3–11 (*p* > 0.05) [23]. Yang et al. reported that MDLFTE and WPPD from protein hydrolysate of *Tergillarca granosa* could not stand the high-temperature (>80 °C) and strong basic (pH > 9.0) processing [25]. Similarly, Jang et al. reported that ATSHH from hydrolysate of sandfish incubated at 50–90 °C reduced its partial DPPH· scavenging activity. In addition, ATSHH lost some biological activity when it was treated at strong basicity (pH 10–12) or acidity (pH 2) [46]. Our results indicate that SMP-3 (PELDW), SMP-7 (WPDHW), SMP-10 (FGYDWW), and SMP-11 (YLHFW) had similar thermal and pH stability with MDLFTE, WPPD, and ATSHH because they could only keep their high activity when they were treated under a low temperature (<60 °C) and a moderate pH environment (pH 5–9).

## 3. Experimental Section

### 3.1. Materials

Spanish mackerel (*S. niphonius*) was purchased from Fengmao Market in Zhoushan city of China. DEAE-52 cellulose and Sephadex G-15 were purchased from Shanghai Source Poly Biological Technology Co., Ltd. (Shanghai, China). Acetonitrile (ACN) and trifluoroacetic acid (TFA) were purchased from Thermo Fisher Scientific Co., Ltd. (Shanghai, China). DPPH and bovine serum albumin (BSA) were purchased from Sigma Aldrich Trading Co., Ltd. (Shanghai, China). Plasmid DNA (pBR322DNA) was purchased from TaKaRa Biotechnology Co., Ltd. (Dalian, China). SMP-3 (PELDW), SMP-7 (WPDHW), SMP-10 (FGYDWW), and SMP-11 (YLHFW) were synthesized in China Peptides Co., Ltd. (Suzhou, China) and used to evaluate their antioxidant activity and stability.

### 3.2. Preparation of Protein Hydrolysate from Spanish Mackerel Muscle

The Spanish mackerel muscle was homogenized and blended with isopropanol at a ratio of 1:4 (*w*/*v*) and stand at 30 ± 2 °C for 6 h, and the isopropanol was changed each 2.0 h. Finally, the solution was filtered using a cheesecloth and the solid precipitate was air-dried at 35 ± 2 °C.

The hydrolytic process of the defatted muscle using five proteases was performed following the previous methods [9]. The dispersions of the defatted muscle (1%, *w*/*v*) were ultrasonic for 15 min and hydrolyzed separately on their optimal hydrolysis parameters (Table 4).

The hydrolytic process of the defatted muscle using in vitro gastrointestinal (GI) digestion was performed on the method described by Yang et al. [10]. Briefly, the defatted muscle powders dispersed in DW (pH 1.5, 1%) were ultrasonic for 15 min and firstly hydrolyzed by pepsin with a dosage of 1 g pepsin/100 g defatted powder under the conditions of 37.0 ± 2 °C and pH 1.5. Two hours later, the pH of the degraded solution was adjusted to 7.0 using a 1.0 M NaOH solution and further hydrolyzed using trypsin with a dosage of 1 g trypsin/100 g defatted powder for 2 h.

After 4 h of hydrolysis, the hydrolysis solutions were heated at 90 ± 2 °C for 20 min and centrifuged at 8000 *g* for 25 min at −4 °C. The resulting supernatant were freeze-dried and kept at −20 °C. The dispersions of the defatted muscle (1%, *w*/*v*) were ultrasonic for 15 min and freeze-dried, and the freeze-dried powder was referred to as SMP. The protein hydrolysate of Spanish mackerel muscle prepared using in vitro GI digestion method was referred to as SMPH. The concentrations of hydrolysates and their fractions were expressed as mg protein/mL and measured by the dye binding method of Bradford [47], and BSA was used as the standard protein.

### 3.3. Isolation of APs from SMPH

#### 3.3.1. Fractionation of SMPH

**Figure 11:**
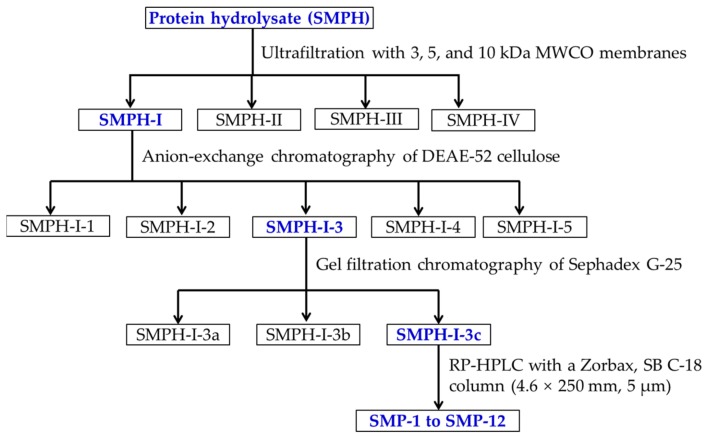
The flow diagram of isolating APs from the hydrolysate (SMPH) of Spanish mackerel muscle prepared using in vitro GI digestion.

Figure 11 shows the flow diagram of isolating APs from SMPH. SMPH was fractionated using ultrafiltration with 3, 5, and 10 kDa MWCO membranes (Millipore, Hangzhou, China), and four fractions termed SMPH-I (MW < 3 kDa), SMPH-II (3 kDa < MW < 5 kDa), SMPH-III (5 kDa < MW < 10 kDa), and SMPH-IV (>10 kDa) were collected and lyophilized.

#### 3.3.2. Chromatography Isolation of APs from SMPH-I

The chromatography isolation process was performed according to previous methods [48,49]. Five milliliters of an SMPH-I solution (40.0 mg/mL) were injected into a pre-equilibrated DEAE-52 cellulose column (1.6 × 80 cm) and separately eluted with 150 mL of DW, 0.1 M NaCl, 0.5 M NaCl, and 1.0 M NaCl solution at a flow rate of 1.0 mL/min. Each eluate (5.0 mL) was collected and detected at 214 nm. Finally, five fractions (SMPH-I-1 to SMPH-I-5) were prepared on the chromatogram. Five milliliters of SMPH-I-3 were separated on a column of Sephadex G-25 (2.6 × 160 cm) eluted with DW at a flow rate of 0.6 mL/min. Each eluate (3.0 mL) was collected and measured at 214 nm. Three fractions (SMPH-I-3a to SMPH-I-3c) were prepared on the chromatogram. Five milliliters of SMPH-I-3c were further purified using RP-HPLC with a Zorbax, SB C-18 column (4.6 × 250 mm, 5 µm) on an Agilent 1260 (Santa Rosa, CA, USA). The sample was eluated with a linear gradient of ACN (0–40% in 0–30 min) in 0.1% TFA at a flow rate of 1.0 mL/min. Twelve major peaks (SMP-1 to SMP-12) were isolated on the absorbance at 214 nm.

### 3.4. Analysis of Amino Acid Sequence and Molecular Mass

The amino acid sequences of four APs (SMP-3, SMP-7, SMP-10, and SMP-11) were measured on an Applied Biosystems 494 protein sequencer (Perkin Elmer/Applied Biosystems Inc, Foster City, CA, USA). The molecular masses of four APs (SMP-3, SMP-7, SMP-10, and SMP-11) were measured using a Q-TOF mass spectrometer coupled with an ESI source (Waters, Los Angeles, CA, USA), respectively.

### 3.5. Antioxidant Activity

#### 3.5.1. Radical Scavenging Assays

The DPPH·, HO·, and $O_{2}^{-}$· scavenging assays of four APs (SMP-3, SMP-7, SMP-10, and SMP-11) were performed according to the previous methods [28,31], and the EC_50_ was defined as the concentration where a sample caused a 50% decrease of the initial radical concentration.

##### DPPH· Scavenging Assay

Two milliliters of samples consisting of DW and different concentrations of the analytes were placed in cuvettes, and 500 μL of an ethanolic solution of DPPH (0.02%) and 1.0 mL of ethanol were added. A control sample containing the DPPH solution without the sample was also prepared. In the blank, the DPPH solution was substituted with ethanol. The DPPH· scavenging activity was calculated using the following formula:

DPPH· scavenging activity (%) = (A_c_ + A_b_ − A_s_)/A_c_ × 100%

where A_s_ is the absorbance rate of the sample, A_c_ is the control group absorbance, and A_b_ is the blank absorbance.

##### HO· Scavenging Assay

One milliliter of a 1.865 mM 1,10-phenanthroline solution and 2.0 mL of the sample were added to a screw-capped tube and mixed. Afterwards, 1.0 mL of a FeSO_4_·7H_2_O solution (1.865 mM) was added to the mixture. The reaction was initiated by adding 1.0 mL of H_2_O_2_ (0.03%, *v*/*v*). After incubating at 37 °C for 60 min in a water bath, the absorbance of the reaction mixture was measured at 536 nm against a reagent blank. The reaction mixture without any antioxidant was used as the negative control, and a mixture without H_2_O_2_ was used as the blank. The HO· scavenging activity was calculated using the following formula:

HO· scavenging activity (%) = [(A_s_ − A_n_)/(A_b_ − A_n_)] × 100%

where A_s_, A_n_, and A_b_ are the absorbance values determined at 536 nm of the sample, the negative control, and the blank after the reaction, respectively.

##### $O_{2}^{-}$· Scavenging Assay

Superoxide anions were generated in 1.0 mL of nitrotetrazolium blue chloride (NBT) (2.52 mM), 1.0 mL of NADH (624 mM), and 1.0 mL of different sample concentrations. The reaction was initiated by adding 1.0 mL of phenazine methosulphate (PMS) solution (120 μM) to the reaction mixture. The absorbance was measured at 560 nm against the corresponding blank after 5 min incubation at 25 °C. The $O_{2}^{-}$· scavenging activity was calculated using the following equation:

$$O_{2}^{-}\cdot \mathrm{scavenging}\;\mathrm{activity}\;(\%)=[(A_{c}-A_{s})/A_{c}]\times 100\%$$

where A_c_ is the absorbance without sample, and A_s_ is the absorbance with sample.

#### 3.5.2. Lipid Peroxidation Inhibition Assay

The lipid peroxidation inhibition activity of the APs was measured in the linoleic acid model system according to the method of Wang et al. [28]. Briefly, a sample (5.0 mg) was dissolved in 10.0 mL of 50.0 mM phosphate buffer solution (PBS, pH 7.0) and added to 0.13 mL of a solution of linoleic acid and 10.0 mL of 99.5% ethanol. The total volume was adjusted to 25 mL with DW. The mixture was incubated in a conical flask with a screw cap at 40 °C in a dark room, and the degree of oxidation was evaluated by measuring ferric thiocyanate values. The reaction solution (100 μL) incubated in the linoleic acid model system was mixed with 4.7 mL of 75% ethanol, 0.1 mL of 30% ammonium thiocyanate, and 0.1 mL of 20 mM ferrous chloride solution in 3.5% HCl. After 3 min, the thiocyanate value was measured at 500 nm following color development with FeCl_2_ and thiocyanate at different intervals during the incubation period at 40 °C.

#### 3.5.3. Protective Effect on Plasmid DNA

The protective effects of four APs (SMP-3, SMP-7, SMP-10, and SMP-11) on supercoiled plasmid DNA (pBR322) were measured according to the previous method [12]. In brief, 15 µL of reaction mixtures containing 5 µL of PBS (10 mM, pH 7.4), 2 µL of FeSO_4_ (1.0 mM), 1 µL of pBR322 (0.5 µg), 5 µL of the AP (SMP-3, SMP-7, SMP-10, or SMP-11, respectively), and 2 µL of H_2_O_2_ (1.0 mM) were incubated at 37 °C. After 0.5 h of incubation, 2 µL of loading buffer containing glycerol (50%, *v*/*v*), EDTA (40 mM), and bromophenol blue (0.05%) were added to terminate the reaction. The resulted reaction mixtures were subsequently electrophoresed on 1% agarose gel containing 0.5 µg/mL EtBr for 50 min (60 V), and the DNA in the agarose gel was photographed under ultraviolet light.

### 3.6. Stability Properties

The stability of four APs (SMP-3, SMP-7, SMP-10, and SMP-11) were measured according to the previous methods [10,50]. The thermostability of four APs (SMP-3, SMP-7, SMP-10, and SMP-11) was determined using a water bath at 20, 40, 60, 80, or 100 °C for 0.5 h. pH values of 3, 5, 7, 9, or 11 were used to evaluate the pH stability of four APs (SMP-3, SMP-7, SMP-10, and SMP-11) at 25 °C for 2.5 h. HO· scavenging activities (EC_50_ value) of the treated four APs (SMP-3, SMP-7, SMP-10, and SMP-11) were measured according to the methods described in Section 2.5.

### 3.7. Statistical Analysis

The data are expressed as the mean ± SD (*n* = 3). ANOVA test for differences between means of each group was used to analyze data using SPSS 19.0 (SPSS Corporation, Chicago, IL, USA). A *p*-value of less than 0.05 was considered statistically significant.

## 4. Conclusions

In the experiment, the proteins of Spanish mackerel (*S. niphonius*) muscle were hydrolyzed under five kinds of enzymes and in vitro GI digestion, and four APs (SMP-3, SMP-7, SMP-10, and SMP-11) were isolated from the hydrolysate prepared using in vitro GI digestion and identified as PELDW, WPDHW, FGYDWW, and YLHFW, respectively. PELDW, WPDHW, FGYDWW, and YLHFW showed high radical scavenging activity, lipid peroxidation inhibition ability, and protective effects on plasmid DNA (pBR322DNA) against oxidative damage induced by H_2_O_2_. Moreover, four APs (PELDW, WPDHW, FGYDWW, and YLHFW) from protein hydrolysate of Spanish mackerel muscle might be applied as an ingredient in new functional foods and products under a normal temperature (<40 °C) and a moderate pH environment (pH 5–9).

## Figures and Tables

**Table 1:** Degree of hydrolysis (%) and DPPH· scavenging activity (%) of protein hydrolysate of Spanish mackerel (*S. niphonius*) muscle using five kinds of enzymes and in vitro GI digestion.

Protease	Degree of Hydrolysis (%)	DPPH· Scavenging Activity (10.0 mg protein/mL, %)
Pepsin	16.58 ± 0.94 ^a^	27.64 ± 1.48 ^a^
Neutrase	20.48 ± 1.62 ^b^	34.28 ± 1.37 ^b^
Papain	17.29 ± 0.48 ^a^	25.98 ± 1.55 ^a^
Trypsin	20.12 ± 1.15 ^b^	32.96 ± 2.33 ^b^
Alcalase	23.47 ± 1.51 ^c^	41.53 ± 3.41 ^c^
in vitro gastrointestinal digestion	26.58 ± 1.25 ^d^	52.58 ± 2.68 ^d^

All data are presented as the mean ± standard deviation (SD, *n* = 3). ^a–c^ Values with the same letters in each column indicate no significant difference (*p* > 0.05).

**Table 2:** Retention time, amino acid sequences, and molecular weights of four isolated peptides (SMP-3, SMP-7, SMP-10, and SMP-11) from protein hydrolysate of Spanish mackerel (*S. niphonius*) muscle.

No.	Retention Time (min)	Amino Acid Sequence	Theoretical Mass/Observed Mass (Da)
SMP-3	11.02	PELDW	658.70/658.72
SMP-7	14.74	WPDHW	739.78/739.81
SMP-10	17.58	FGYDWW	872.92/872.93
SMP-11	19.83	YLHFW	764.87/764.90

**Table 3:** EC_50_ vales of four APs (SMP-3, SMP-7, SMP-10, and SMP-11) and the positive control of glutathione (GSH) on DPPH·, HO·, and O·.

No.	Half Elimination Ratio (EC_50_, mg/mL)		
	DPPH·	HO·	$O_{2}^{-}$·
SMP3	1.53 ± 0.12 ^a^	1.12 ± 0.09 ^a^	0.85 ± 0.07 ^a^
SMP7	0.70 ± 0.04 ^b^	0.38 ± 0.02 ^b^	0.49 ± 0.04 ^b^
SMP10	0.53 ± 0.03 ^c^	0.26 ± 0.02 ^c^	0.34 ± 0.05 ^c^
SMP11	0.97 ± 0.06 ^d^	0.67 ± 0.05 ^d^	1.37 ± 0.11 ^d^
GSH	0.22 ± 0.01 ^e^	0.12 ± 0.01 ^e^	0.09 ± 0.01 ^e^

All data are presented as the mean ± SD (*n* = 3). ^a–f^ Values with the same letters indicate no significant difference of different samples at the same radicals (*p* > 0.05).

**Table 4:** Hydrolysis parameters of different proteases and their combination.

Protease	Temperature (°C)	Enzyme Dosage (g Enzyme/100 g Defatted Muscle)	Time (h)	pH Value
Pepsin	37	2	4	2.0
Neutrase	60	2	4	7.0
Papain	50	2	4	6.0
Trypsin	37	2	4	7.0
Alcalase	50	2	4	8.0
In vitro gastrointestinal digestion	37	Trypsin 1	2	2.0
		Pepsin 1	2	7.0
